# Differential expression of CPKs and cytosolic Ca^2+^ variation in resistant and susceptible apple cultivars (*Malus x domestica*) in response to the pathogen *Erwinia amylovora* and mechanical wounding

**DOI:** 10.1186/1471-2164-14-760

**Published:** 2013-11-05

**Authors:** Chidananda Nagamangala Kanchiswamy, Tapan Kumar Mohanta, Andrea Capuzzo, Andrea Occhipinti, Francesca Verrillo, Massimo E Maffei, Mickael Malnoy

**Affiliations:** 1 Research and Innovation Centre Genomics and Biology of Fruit Crop Department, Fondazione Edmund Mach (FEM), Istituto Agrario San Michele (IASMA), Via Mach 1, 38010, San Michele all’Adige (TN), Italy; 2 Department of Life Sciences and Systems Biology, Innovation Centre, University of Turin, Via Quarello 15/A, Turin 10135, Italy

**Keywords:** *Malus* x domestica, *Calcium dependent Protein Kinases*, *Erwinia amylovora*, Phylogenetic analysis, Gene expression, Cytosolic calcium variations

## Abstract

**Background:**

Plant calcium (Ca^2+^) signals are involved in a wide array of intracellular signalling pathways following pathogen invasion. Ca^2+^-binding sensory proteins such as Ca^2+^-dependent protein kinases (CPKs) have been predicted to mediate signalling following Ca^2+^ influx after pathogen infection. However, to date this prediction has remained elusive.

**Results:**

We conducted a genome-wide identification of the *Malus x domestica CPK* (*MdCPK*) gene family and identified 30 *CPK* genes. Comparative phylogenetic analysis of *Malus* CPKs with CPKs of *Arabidopsis thaliana* (*AtCPKs*), *Oryza sativa* (*OsCPKs*), *Populous trichocarpa* (*PtCPKs*) and *Zea mays* (*ZmCPKs*) revealed four different groups. From the phylogenetic tree, we found that *MdCPKs* are closely related to *AtCPKs* and *PtCPKs* rather than *OsCPKs* and *ZmCPKs*, indicating their dicot-specific origin. Furthermore, comparative quantitative real time PCR and intracellular cytosolic calcium ([Ca^2+^]_cyt_) analysis were carried out on fire blight resistant and susceptible *M*. x *domestica* apple cultivars following infection with a pathogen (*Erwinia amylovora*) and/or mechanical damage. Calcium analysis showed an increased [Ca^2+^]_cyt_ over time in resistant cultivars as compared to susceptible cultivars. Gene expression studies showed that 11 out of the 30 *MdCPKs* were differentially expressed following pathogen infection.

**Conclusions:**

We studied the genome-wide analysis of *MdCPK* gene family in *Malus* x *domestica* and analyzed their differential gene expression along with cytosolic calcium variation upon pathogen infection. There was a striking difference in *MdCPKs* gene expressions and [Ca^2+^]_cyt_ variations between resistant and susceptible *M*. *x domestica* cultivars in response to *E*. *amylovora* and mechanical wounding. Our genomic and bioinformatic analysis provided an important insight about the role of *MdCPKs* in modulating defence responses in susceptible and resistant apple cultivars. It also provided further information on early signalling and downstream signalling cascades in response to pathogenic and mechanical stress.

## Background

Calcium ions (Ca^2+^) plays a central role as a second messenger in nearly every aspect of cellular signalling. In plants, regulation of cytosolic Ca^2+^-concentration ([Ca^2+^]_cyt_) occurs in response to various endogenous and external signals, including changes in hormone status, abiotic stress stimuli such as drought, high and low temperature or light, biotic stress stimuli such as pathogen infection, microbial elicitors and symbiotic nodulation factors, as well as mechanical wounding [1-4]. Ca^2+^ influx is also a prerequisite for programmed cell death in plants [5,6]. These Ca^2+^ signatures are perceived by different Ca^2+^ sensor molecules which subsequently transduce the signal to downstream signalling cascades such as phosphorylation of target proteins [3,7,8].

Plants have four different classes of Ca^2+^ sensors: clamodulins (*CaM*), clamodulin-like proteins (*CaML*), calcineurin B-like proteins (*CBL*) and calcium-dependent protein kinases (*CPKs*) [9]. *CaM*, *CaML* and *CBL* lack an effector domain and contain only a Ca^2+^ binding domain; hence, they can sense and transmit Ca^2+^ signals by interacting with target proteins [10]. In *Arabidopsis*, the *CaM*-like protein (*CML24*) is required for nitric oxide (NO) production and *AvrRpt2*-mediated programmed cell death (PCD) [5], whereas *CML42*-mediated Ca^2+^ signalling coordinates responses to herbivory and abiotic stress [11].

*CPKs* constitute a large family of serine/threonine protein kinases that are widely distributed in the plant kingdom. For example, the *Arabidopsis* genome is predicted to have 34 different *CPKs*, *Zea mays* has 34, *Populus* 30, *Oryza* 31 and *Triticum* 24 *CPKs*[9,12-14], which can be classified into four groups (I-IV) based on sequence similarity [15]. *CPKs* are of special interest, since they represent a new class of Ca^2+^ sensors, having both a protein kinase domain and a calmodulin-like domain (including an EF-hand calcium-binding site) in a single polypeptide [9,15]. *CPKs* are activated by the binding of Ca^2+^ at the EF-motifs, resulting in protein conformational changes that in turn drive the auto inhibitory domain to become detached from the protein kinase domain [16]. Activated *CPKs* can mediate Ca^2+^ signalling by phosphorylating their substrate proteins [3]. The *N*- and *C*-terminal domains are variable, differing in their length and amino acid composition in various *CPK* proteins and it has been suggested that these variable domains determine the specific functions of individual *CPKs*[17,18]. *Arabidopsis CPK1* was the first CPK to be characterised, and is known to be activated by phospholipids and 14-3-3 proteins [19]. *AtCPKs* 3, 4, 6, 11 and 32 act as abscisic acid (ABA) signalling components, and are involved in ABA-responsive gene expression, seed germination, seedling growth, and stomatal movement [20-22]. In plant immunity, four *Arabidopsis CPKs* (*CPKs* 4/5/6/11) have been shown to play important roles, together with mitogen activated protein kinase (*MAPK*) cascades, in relaying primary microbe associated molecular pattern (MAMP) immune signalling [23]. Recently, six *Arabidopsis CPKs* have been identified in sensing and transducing Ca^2+^, indicating the specificity and redundancy of individual *CPKs* in nucleotide-binding domain leucine-rich repeat (NLR) signalling events: *CPK4*/*5*/*6*/*11* modulate immune response expression, *CPK1*/*2*/*4*/*11* ROS production, and *CPK1*/*2*/*5*/*6* are involved in programmed cell death (PCD), as revealed by integrative molecular analyses [6,24]. Apparently, specific *CPKs* are engaged in diverse immune responses via phosphorylation and activation of *WRKY* transcription factors. For example, activation of *CPK4*/*5*/*6*/*11* phosphorylates *WRKY8*/*28*/*48* for transcription reprogramming of immune genes; *CPK1*/*2*/*4*/*11* phosphorylates *NADPH oxidases* for ROS production and contributes to PCD [6]. These results indicate that *CPKs* are involved in the bifurcation of NLR signalling mechanisms.

The most economically important fruit and ornamental trees and bushes, such as apple (*Malus* × *domestica*), pear (*Pyrus communis*), peach (*Prunus persica*), cherry (*Prunus avium*), strawberry (*Fragaria* spp.), apricot (*Prunus armeniaca*), almond (*Prunus amygdalus*) and rose (*Rosa hybrida*) all belong to the Rosaceae family [25]. *M*. × *domestica* is one of the most economically important woody plants cultivated worldwide as a fruit crop, however the function of apple *CPKs* in the immune response to pathogens has never been reported.

The enterobacterial phytopathogen *Erwinia amylovora* causes fire blight, an invasive disease that threatens a wide range of commercial and ornamental Rosaceae host plants [26]. It has been difficult to eradicate or reduce the incidence of fire blight epidemics. Management practices include the use of a few size-controlling rootstocks that are resistant to fire blight and chemical treatments to enhance host resistance [26]. Molecular investigations of the *E*. *amylovora*-*Malus* interaction have been limited to a restricted number of plant defences previously characterised in other plant-pathogen interactions [27], or via untargeted analysis [28-31]. These different molecular approaches have provided a long list of up or down regulated genes in susceptible or resistant plants, but have not elucidated the mechanism of apple susceptibility or resistance to fire blight.

Here we undertook a genome wide study to identify and to classify the *CPKs* involved in the defence response of *M*. × *domestica* against the pathogen *E*. *amylovora*. A gene encoding *CPK* was shown to be up-regulated in the blossom of susceptible apple cultivars after *E*. *amylovora* infection, suggesting that Ca^2+^ may be one of the key signals that initiates stress resistance reactions in blossom [31]. In order to identify genes implicated in the control of fire blight resistance, we evaluated [Ca^2+^]_cyt,_ the role of *CPKs* in early signalling cascades in the cultivars Golden delicious 'GD' (susceptible) and 'M.7' (resistant) [28] following challenge with a virulent strain of *E*. *amylovora* (Ea273) or mechanical damage.

The purpose of this study was to understand the mechanisms of interaction between *M*. × *domestica* and *E*. *amylovora* in resistant and susceptible apple cultivars. The results will help to design new strategies to improve apple resistance to *E*. *amylovora* and facilitate development of resistant transgenic lines for economically important susceptible cultivars.

## Results

### *MdCPK* gene family is distributed in 14 out of 17 chromosomes

**Figure 1:**
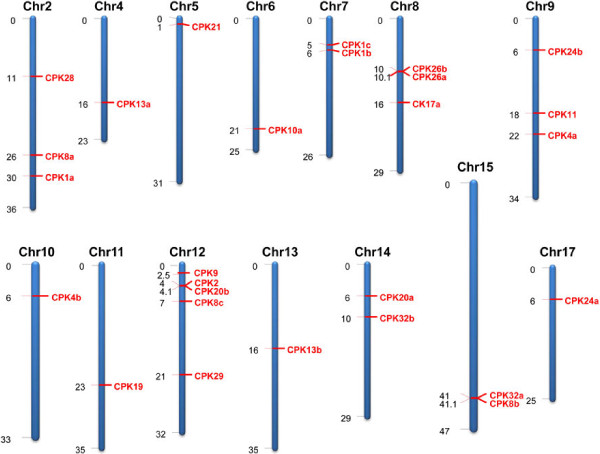
**Genomic distribution of *MdCPK *genes in *Malus *chromosomes.** The number in brackets shows the position of the gene on the *Malus* chromosome pseudomolecules. The chromosome numbers are indicated at the top of each bar. Figure show, *MdCPK* genes are distributed evenly in different chromosome.

*M*. × *domestica* has a diploid genome that underwent a whole genome duplication for 50 million years ago. It has x = 17 chromosomes containing 26,374 loci and 63,541 transcripts, organised in a 881.3 Mb genome [32,33]. A genome-wide search for memebrs of the *MdCPK* gene family led to identification of 30 *CPK* genes. Most of the *CPK* genes have alternative transcript variants. *MdCPK11* has 15 possible transcript variants (Table 1). Transcript organisation of *MdCPKs* shows an average of 8 exons per gene, with the exception of *MdCPK11*, which has no introns (Table 1, Additional file 1: Figure S1). *CPK* genes are distributed in 14 of the 17 apple chromosomes (Figure 1). Most *CPK* genes are present in clusters rather than displaying tandem distribution. Chromosome 12 contains five *MdCPKs* (*MdCPK2*, *MdCPK8c*, *MdCPK9*, *MdCPK20b*, and *MdCPK29*) whereas, chromosome 8 and 9 contain three *CPKs* (*MdCPK17a*, *MdCPK26a*, *MdCPK26b* and MdCPK4a, *MdCPK11*, *MdCPK24b* respectively).

**Table 1:** **Phytozome locus ID and transcript details of *Malus CPKs***

**Sl.No**	**Phytozome locus ID**	**Location in chromosome**	**Gene name**	**ORF**	**No.of a.a**	**No.of Introns**	**No.of alternative splicing variants**	**5′-3′Coordinates**	**Phylogenetic group**
1	MDP0000153100	2	MdCPK1a	1694	566	6	5	MDC017159.84: 8453 - 14350	I
2	MDP0000142687	7	MdCPK1b	1763	618	8	5	MDC021045.283: 1756 - 8958	I
3	MDP0000128057	7	MdCPK1c	1943	660	8	5	MDC013839.354: 42 - 7131	I
4	MDP0000232344	12	MdCPK2	2296	775	8	4	MDC012227.366: 34198 - 38360	I
5	MDP0000260834	9	MdCPK4a	1553	517	6	4	MDC020449.143: 14625 - 18251	I
6	MDP0000232885	10	MdCPK4b	1544	518	6	2	MDC010220.255: 18291 - 21903	I
7	MDP0000269423	2	MdCPK8a	1612	553	8	3	MDC001073.515: 2333 - 8854	IV
8	MDP0000119457	15	MdCPK8b	1417	476	6	3	MDC001073.498: 3281 - 6157	IV
9	MDP0000260857	12	MdCPK8c	1881	665	9	5	MDC021346.204: 29191 - 34837	IV
10	MDP0000169895	12	MdCPK9	1451	491	2	4	MDC003603.228: 1126 - 2789	I
11	MDP0000218522	6	MdCPK10a	1692	570	7	5	MDC020438.169: 10660 - 14149	IV
12	MDP0000301254	Unanchored	MdCPK10b	1618	548	7	8	MDC016267.124: 15630 - 19053	IV
13	MDP0000308706	Unanchored	MdCPK10c	1613	548	7	8	MDC020438.160: 35695 - 39116	IV
14	MDP0000494270	9	MdCPK11	1489	498	0	15	MDC010082.361: 3158 - 4654	I
15	MDP0000164868	4	MdCPK13a	1757	585	8	4	MDC000306.525: 1570 - 6773	IV
16	MDP0000649496	13	MdCPK13b	1023	345	4	2	MDC000271.449: 354 - 2825	IV
17	MDP0000802997	8	MdCPK17a	1591	533	7	2	MDC040478.10: 1862 - 4930	II
18	MDP0000138436	Unanchored	MdCPK17b	1605	534	7	3	MDC010071.376: 1022 - 3758	II
19	MDP0000180811	11	MdCPK19	1496	504	9	1	MDC008434.490: 2551 - 5781	II
20	MDP0000318339	14	MdCPK20a	2994	1023	10	3	MDC031256.8: 21258 – 31086	I
21	MDP0000513005	12	MdCPK20b	1963	679	7	0	MDC008272.442: 6235 - 19231	I
22	MDP0000232001	5	MdCPK21	1641	554	7	3	MDC002417.261: 24324 - 28052	II
23	MDP0000262701	17	MdCPK24a	1623	541	7	3	MDC020007.246: 24451 - 27032	IV
24	MDP0000282003	9	MdCPK24b	2860	954	12	2	MDC006465.419: 8202 - 16334	IV
25	MDP0000297184	8	MdCPK26a	1685	571	6	3	MDC012276.352: 7244 - 10346	I
26	MDP0000457940	8	MdCPK26b	4152	1403	8	0	MDC001323.383: 1559 - 7846	I
27	MDP0000208913	2	MdCPK28	1861	626	13	10	MDC018730.149: 4526 - 9378	III
28	MDP0000142398	12	MdCPK29	1584	527	7	4	MDC015573.110: 52421 - 55302	II
29	MDP0000649508	15	MdCPK32a	2081	709	10	4	MDC001801.279: 799 - 8644	IV
30	MDP0000179069	14	MdCPK32b	2011	676	10	3	MDC006959.379: 1716 - 6520	IV

### Phylogenetic analysis shows that *MdCPKs* are clustered into four clades

**Figure 2:**
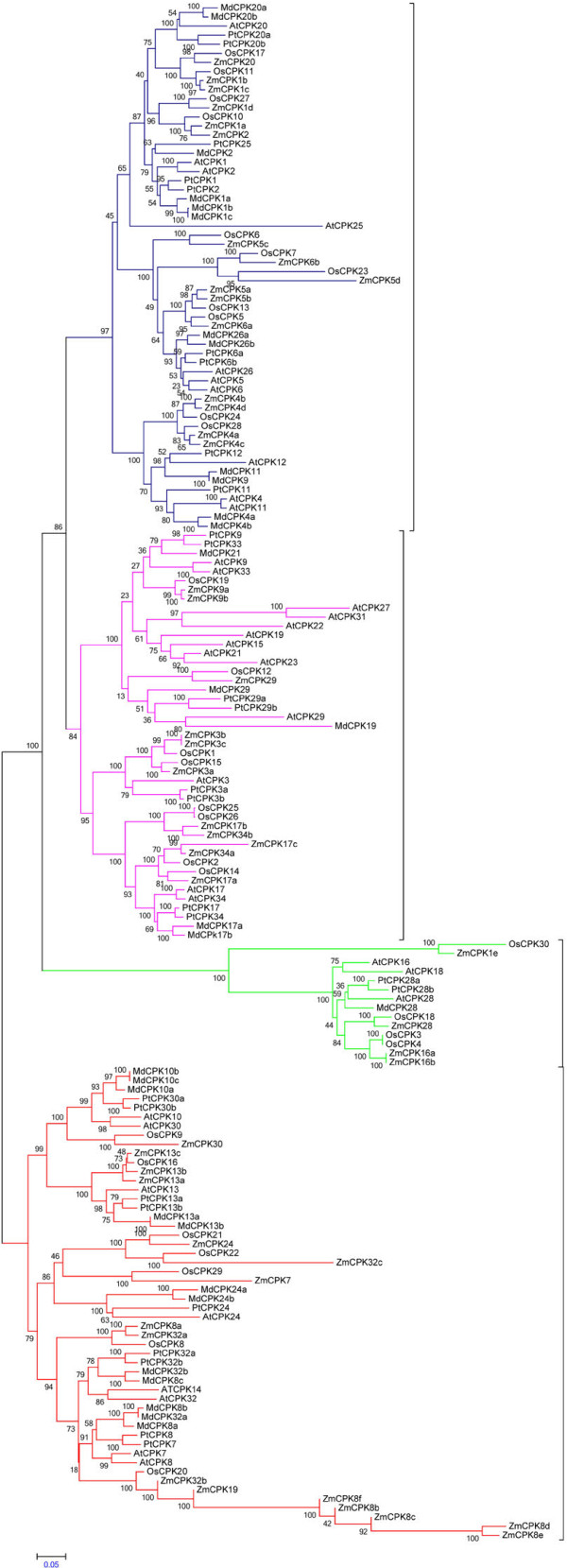
**Phylogenetic tress of Malus *CPKs *with orthologous *CPKs *of *Arabidopsis thaliana *(*AtCPKs*), *Oryza sativa *(*OsCPKs*), *Populous trichocarpa *(*PtCPKs*) and *Zea mays *(*ZmCPKs*).** Phylogenetic trees show, all the *CPKs* are clustered into four different groups and *MdCPKs* genes are found to be much close to *AtCPKs*. Phylogenetic tree was constructed by MEGA5 software and statistical method used was Neighbor-joining, substitutition type: amino acid, Model: Jones-Taylor-Thornton (JTT) and no. of bootstrap replication was 500.

The *Malus MdCPK* amino acid sequence length ranged from 345 (*MdCPK13b*) to 1403 amino acids (*MdCPK26b*). Cluster analysis identified thirty *MdCPKs* nested into four distinct clades, as shown in Figure 2. A phylogenetic study of *MdCPKs* with orthologous *A*. *thaliana*, *O*. *sativa*, *P*. *trichocarpa and Z*. *mays* also clustered into four clades. MdCPKs are closely related to *AtCPK* and *PtCPKs* and that the proposed nomenclature for *MdCPKs* is consistent. The *OsCPKs* and *ZmCPKs* are less closely related to *MdCPKs* indicating their dicot-specific origin (Figure 2).

### All *MdCPKs* have an EF-hand domain and palmitoylation sites

Ca^2+^ signals are decoded by many different protein kinases, and *CPKs* play significant roles in these signalling events [24,34,35]. The Ca^2+^ binding EF-hands are the predominant Ca^2+^ sensors, consisting of twelve residue loops, flanked on both sides by twelve alpha-helical domain residues [Additional file 2: Figure S2].

In response to *E*. *amylovora* and mechanical damage, *MdCPKs* are differentially expressed in resistant 'M.7' and susceptible 'G.D' *M*. *x domestica* cultivars

**Figure 3:**
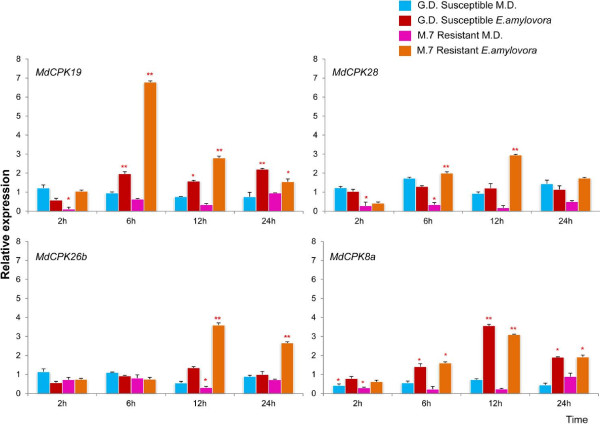
**Quantitative RT**-**PCR comparison of resistant and susceptible *Malus *cultivars after *E***. ***amylovora *infection and mechanical damage at 2, 6 12 and 24 hpi.** The transcript level of genes in resistant/susceptible cultivars at 2,6,12 and 24 hpi were normalised with those of *EF1* and *UB1* measured in the samples and expressed in relation to the normalised transcript level in the leaves of the respective uninfected plants (0 hrs). Metric bars represent the standard error (SE). Asterisks indicate significant differences: * *P* < 0.05, ** *P* < 0.01.

To clarify *MdCPKs* role in the resistance and susceptibility of *M*. × *domestica* to *E*. *amylovora* and mechanical damage (MD), we carried out a comparison between *CPK* gene expression patterns in the resistant Malling7 apple rootstock (M.7) and the susceptible golden delicious (GD) by using quantitative real time PCR analysis (qPCR) at 2, 6, 12 and 24 hours post inoculation (hpi) (Figure 3). These time points were selected based on previous analyses of the temporal transcriptional response of *Malus* to *E*. *amylovora*, indicating that basal defence to pathogen associated molecular patterns (PAMPs) occurred within 1–2 hpi, whereas expression of defence proteins occurred at 24–48 hpi [28]. These two genotypes were chosen based on their level of resistance and susceptibility to fire blight disease. M.7 is a highly resistant genotype whereas GD is a susceptible genotype to fire blight disease.

**Figure 4:**
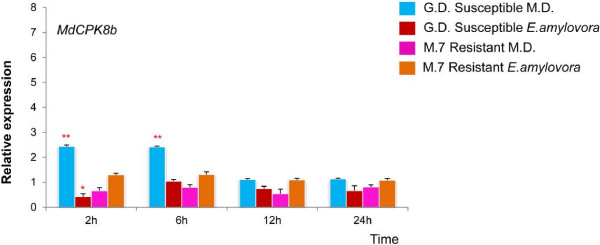
**Quantitative RT**-**PCR comparison of resistant and susceptible *Malus *cultivars after *E***. ***amylovora *infection and mechanical damage at 2, 6, 12 and 24 hpi.** The transcript level of genes in resistant/susceptible cultivars at 2,6,12 and 24 hpi were normalised with those of *EF1* and *UB1* measured in the samples and expressed in relation to the normalised transcript level in the leaves of the respective uninfected plants (0 hrs).Metric bars represent the standard error (SE). Asterisks indicate significant differences: * P < 0.05, ** P < 0.01.

**Figure 5:**
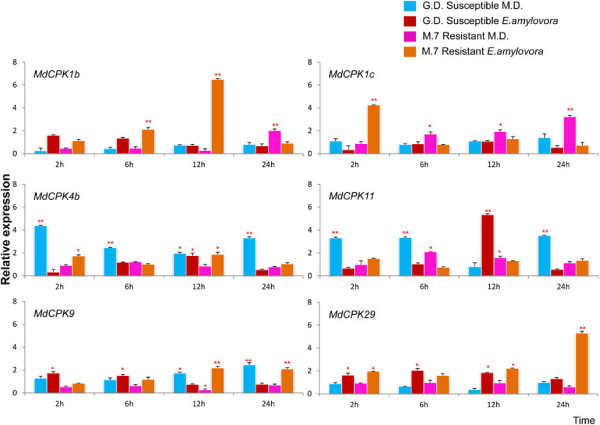
**Quantitative RT**-**PCR comparison of resistant and susceptible *Malus *cultivars after *E***. ***amylovora *infection and mechanical damage at 2, 6, 12 and 24 hpi.** The transcript level of the genes in resistant/susceptible at 2,6,12 and 24 hpi were normalised with those of *EF1* and *UB1* measured in the samples and expressed in relation to the normalised transcript level in the leaves of the respective uninfected plants (0 hrs). Metric bars represent the standard error (SE). Asterisks indicate significant differences: * P < 0.05, ** P < 0.01.

Few of the *MdCPKs* were up-regulated in the M.7 resistant genotype as compared to GD susceptible plants. Of the thirty *MdCPKs* analysed by qPCR, only eleven showed differential expression in susceptible and resistant apple genotypes following *E*. *amylovora* infection or MD (Figures 3, 4 and 5).

Four *MdCPK*s were specifically induced after infection with *E*. *amylovora* (Figure 3). In the resistant genotype following *E*. *amylovora* infection, *MdCPK19* and *MdCPK28* were significantly up regulated at 6 and 12 hpi, whereas *MdCPK26b* was up regulated at 12 and 24 hpi. Following *E*. *amylovora* infection, *MdCPK8a* was similarly up regulated at 6, 12 and 24 hpi in both resistant and susceptible cultivars. None of the *CPK* genes were activated after MD (Figure 3). However, *MdCPK8b* was specifically induced in response to MD in the susceptible genotype at 2 and 6 hpi, whereas the resistant genotype showed no induction after either *E*. *amylovora* infection or MD (Figure 4).

Six other *MdCPKs* were differentially expressed in resistant and susceptible cultivars following *E*. *amylovora* infection and/or MD at different time points (Figure 5). In the resistant genotype, four *CPKs* (*MdCPK1b*, *MdCPK1c*, *MdCPK9* and *MdCPK29*) were significantly up regulated at different time points following *E*. *amylovora* infection (Figure 5). It is interesting to note that in response to *E*. *amylovora* infection, *MdCPK1b* was up-regulated at later than *MdCPK1c* and that both genes were up-regulated later following MD than *E*. *amylovora* infection (24 hpi, Figure 5). The susceptible genotype showed a significant up regulation of *MdCPK4b* and *MdCPK11* (except for 12 hpi) following MD (Figure 5).

*E*. *amylovora* induced differential intracellular cytosolic calcium variations in susceptible and resistant *M*. x *domestica* genotypes

**Figure 6:**
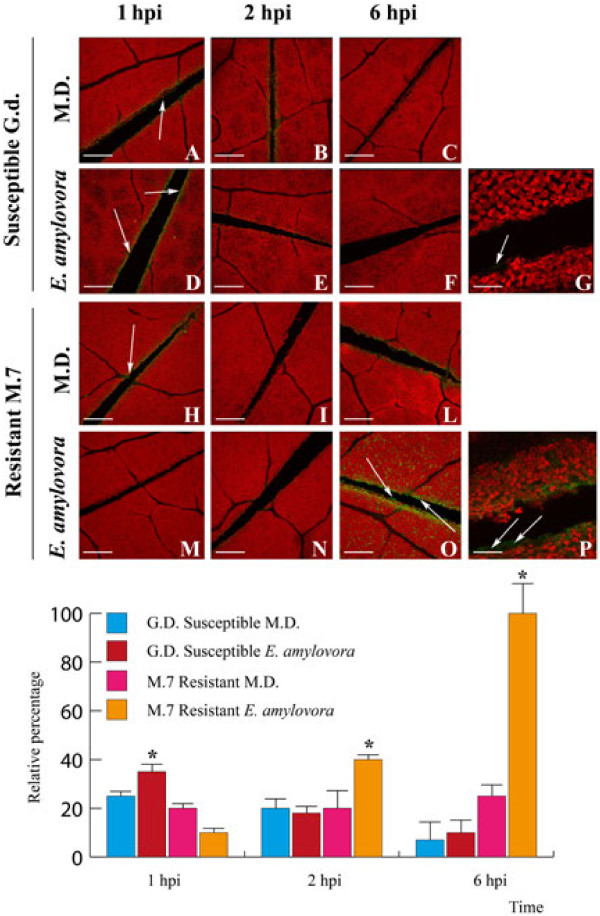
**Intracellular Ca**^**2**+^**variations in GD and *M7 *leaf cells following pathogen infection and mechanical damage.** Leaves were treated with calcium orange for 1 hr and then infected with *E*. *amylovora*. The cytosolic Ca^2+^ concentration of leaf cells was determined 1 hr, 2 hr and 6 hrs after infection. Mechanically damaged (MD) leaves served as a control for both genotypes. In the lower panel, the bar represents the relative percentage of calcium orange fluorescence in at least 3 replicates. Asterisk indicate significant (p <0.05) differences. Scale bars for Figures A-F and H-O = 100 μm, scale bars for Figures G and P = 400 μm.

*CPK* activity is often associated with variations in [Ca^2+^]_cyt_[3,35-38]. Having determined that some *MdCPK* genes are differentially expressed following *E*. *amylovora* infection in resistant and susceptible *M*. × *domestica* cultivars, we evaluated [Ca^2+^]_cyt_ by Confocal Laser Scanning Microscopy (CLSM) with the calcium indicator, calcium orange. In the susceptible genotype, [Ca^2+^]_cyt_ was found to decrease in both MD (Figure 6, A-C) and *E*. *amylovora* infected leaves (Figure 6, D-F) from 1 to 6 hpi. Conversely, the resistant genotype showed a consistent and significant (p < 0.05) increase in [Ca^2+^]_cyt_ over the same time period. In particular, *E*. *amylovora* infected leaves (Figure 6, M-O) showed a higher [Ca^2+^]_cyt_ level when compared to both infected (Figure 6, D-F) and MD resistant genotype leaves (Figure 6, H-L). A higher magnification of resistant genotype leaves at 6 hpi showed a clear cytosolic localization of Ca^2+^ (Figure 6, P), which is more evident than in the susceptible genotype infected leaves (Figure 6, G). Figure 6 also shows the relative percentage of calcium orange fluorescence in both resistant and susceptible apple cultivars upon MD and *E*. *amylovora* infection.

## Discussion

Regulation of Ca^2+^ homeostasis is important, particularly when Ca^2+^ is involved as a signalling ion. In plant cells, Ca^2+^-binding proteins also serve as regulators of internal free Ca^2+^ levels [4,5,38,39]. Since *CPKs* may be involved in the specificity and cross-talk of signal transduction in a variety of biotic and abiotic stresses, their possible involvement in active signalling cascades in response to pathogens deserves a thorough investigation. Recent expression profiles of *M*. × *domestica* blossom–*Erwinia* interaction revealed the involvement of *CPKs* in the signal transduction process [31]. However, a detailed study on the involvement of the *MdCPK* gene family in resistant and susceptible apple plants is lacking.

This work provides fundamental information on the phylogeny, gene structure, and gene expression of *MdCPKs* in response to pathogen and wound signalling in fire blight resistant and susceptible apple cultivars. The *M*. × *domestica* (GD) genome sequence is assembled in 21,554 scaffolds and different gene families reside within these scaffolds. The *CPK* gene family is one of them and is evenly distributed throughout the 17 pseudomolecules of the GD genome sequence. A global survey of the recent apple genome database reveals the presence of 30 *MdCPKs* from 57,386 annotated genes in the apple genome [40]. All the *MdCPKs* analysed here have the typical structures of the *CPK* family, including an *N*-terminal variable domain, a protein kinase domain, an auto-inhibitory domain, a calmodulin-like domain, an EF-hand like domain and a C-terminal domain. The calcium binding EF-hands are the predominant Ca^2+^ sensors.

Comparative plant genomics studies show that plant gene families are largely conserved over evolutionary timescales, including diversification of angiosperm and non-flowering plants [41]. Co-linearity resulting from the common ancestors of the angiosperms provides a powerful way of determining orthology, while comparative sequence analyses provides a wealth of information about the nature of sequence arrangement and evolution [42]. In this study, comparative sequence analysis of the orthologous protein sequences of *Malus CPKs* in relation to *A*. *thaliana* and *P*. *trichocarpa CPKs* showed a high level of conservation and significant orthology compared to *O*. *sativa* and *Z*. *mays CPKs*[12,13]. Improved orthologous gene detection is critically important for accurate functional annotation and study of comparative and evolutionary genomics. Besides this, all the 30 *Malus CPKs* are highly homologous to each other. Furthermore, the similarity found between *MdCPK* gene family with *AtCPK*s shows that *Malus* and *Arabidopsis CPKs* may derive from a common ancestor. Despite this evolutionary conservation of gene families, lineage-specific fluctuations in gene family size are frequent among taxa [41,43].

In this study we found that in group III there is only one *Malus CPK* (*MdCPK28*) present in the phylogenetic tree as compared to three from *Arabidopsis*, four from *Zea*, four from *Oryza* and two from *Populous*. The presence of *MdCPK* (*MdCPK28*) in group III was very divergent from other *MdCPKs* and may have evolved in Rosaceae following divergence with a distinct dicot specific function.

In the EF-hand loop, Ca^2+^ is coordinated in a pentagonal bi-pyramidal configuration [44]. The six residues involved in Ca^2+^ binding are 1, 3, 5, 7, 9 and 12. The invariant Glu (E) or Asp (D) amino acids at position 12 provide two O_2_ that can bind Ca^2+^ ions. The variable *N*-terminal domain contains myristoylation or palmitoylation sites. Palmitoylation is the reversible covalent attachment of palmitic acid to cysteine and less frequently to serine or threonine residues of proteins. Palmitoylation enhances the hydrophobicity of proteins and helps association with membranes (as well as sub-cellular trafficking between membrane compartments) and helps protein-protein interactions [45]. All *MdCPKs* here reported contain palmitoylation sites, usually present at the 4th or 5th position of the *N*-terminal end (Table 2).

**Table 2:** **Prediction of putative palmitoylation sites of *MdCPKs *** **using CSS**-**palm 3**.**0**

**Gene**	**Position**	**Sequence**	**Score**	**Cutoff**
MdCPK1a	5	***MGNTCVGPSISK	1.467	0.196
MdCPK1b	5	***MGNTCVGPSISK	1.576	0.196
MdCPK1c	5	***MGNTCVGPSISK	1.576	0.196
MdCPK2	10	PRDDQIGCQXYLQLS	2.645	1.225
MdCPK4a	37	QFGTTYLCTHKPTGA	0.152	0
MdCPK4b	44	QFGTTYLCTHKPTGA	0.157	0
MdCPK8a	93	EFGVTYLCTEASSNE	0.224	0.196
MdCPK8b	4	****MGNCCVTLGAP	3.132	1.225
MdCPK8c	4	****MGNCCATPQTG	2.814	0.308
MdCPK9	11	KATPSTICSTXASDL	1.43	1.22
MdCPK10a	4	****MGNCNVCVRAD	2.777	1.225
MdCPK10b	4	****MGNCNVCVRAD	3.132	1.225
MdCPK10c	4	****MGNCNVCVRAD	3.132	1.22
MdCPK11	48	QFGTTYLCTEISSGH	0.471	0
MdCPK13a	4	****MGNCCRSPAAV	2.824	0.308
MdCPK13b	28	VILYILLCGVPPFWA	0.219	0.196
MdCPK17a	4	****MGNCCSQCNTE	3.567	0.308
MdCPK17b	4	****MGNCCSQRNTE	4.248	0.308
MdCPK19	139	RGQAVCPCLYGAGEL	0.907	0.497
MdCPK20a	91	ITSRQFVCAHQGKHV	0.357	0.308
MdCPK20b	198	QFGTTFLCVEKETNK	0.31	0.308
MdCPK21	3	*****MGCYSSKENA	2.319	0.308
MdCPK24a	4	****MGSCLCTPANA	0.943	0.308
MdCPK24b	4	****MGSCVCTPAKA	4.019	0.497
MdCPK26a	5	***MGNTCRGSFRGK	2.11	0.308
MdCPK26b	26	IGTPLYLCCRSLTFS	1.657	0.308
MdCPK28	4	****MGICFSAVKVS	4.727	1.225
MdCPK29	4	****MGLCFTKCQSH	1.514	0.308
MdCPK32a	4	****MGNCCVTLGAP	3.132	1.225
MdCPK32b	4	****MGNCCVTPQTG	2.252	0.308

The prediction showed that all *MdCPKs* identified had palmitoylation sites characterised by the presence of cysteine residues at the N-terminal end, usually in positions 4 and 5.

Presence of “****” indicate palmitoylation site present at 4th position and “***” indicate palmitoylation site present at 5th position of respected CPK gene.

In eukaryotes and higher plants, some genes are spliced alternatively during various developmental stages or in response to stresses, creating multiple mRNA transcript for a single gene [46]. Spliced genes may encode proteins with different functions or different cellular or sub-cellular localizations [47]. In this study, *MdCPKs* were found to have several alternative spliced transcript variants (Table 1). The majority of plant alternative spliced transcripts have not yet been functionally characterised, but the evidence suggests that alternative splicing plays a major role in plant function, including stress response, and may impact domestication and trait selection [48]. Splicing variants play important roles within cells and increase proteome diversity and cellular function [49]. Thus, the presence of a significant number of alternative splicing variants in *Malus* might explain its domestication and resistance to stress response. Further studies are necessary to better understand their independent role in different stress responses.

Our study also provides information on the possible involvement of *MdCPKs* in regulating *E*. *amylovora* infection and wound response via Ca^2+^-mediated signalling. The differential expression of Md*CPKs* in fire blight resistant and susceptible *M*. × *domestica* cultivars shows the involvement of *CPKs* in the regulation of *E*. *amylovora* infection and/or to MD The selective expression of a few *CPKs* in the resistant cultivar in response to *E*. *amylovora* indicates the importance of these *CPKs* in modulating the resistance/susceptibility mechanisms by transducing the signal to downstream defence signalling pathways [3,4,38].

The early induction of a few *CPKs* observed, specifically, in the resistant cultivar, indicates they may play an important role in recognising pathogen infection and transducing the signals to downstream signalling cascades. These data show a divergent role for *CPKs* in response to various stimuli and their specific recognition [4,6,50,51].

[Ca^2+^]_cyt_ variations occur in response to various biotic and abiotic stresses [3,4,52-55]. In our study we found that the M.7 resistant cultivar showed a significantly higher [Ca^2+^]_cyt_ accumulation to *E*. *amylovora* infection and MD, whereas the GD susceptible cultivar showed a decreased [Ca^2+^]_cyt_ accumulation. These [Ca^2+^]_cyt_ differences between the M.7 and GD cultivars in response to *E*. *amylovora* infection show the ability of the resistant plant to recognise *E*. *amylovora* infection by significantly inducing [Ca^2+^]_cyt_ accumulation and transducing downstream signalling cascades and are consistent with induction of *MdCPKs* genes. It has been shown that recognition of the pathogen or its effectors increases [Ca^2+^]_cyt_ elevation in plant cells, which is a prerequisite for hypersensitive response development [56-58]. Despite a significant correlation between Ca^2+^ influxes and pathogen recognition, how the Ca^2+^ signal is transduced to downstream signalling events remains elusive. However, recent discoveries have identified six closely related *CPKs* in *Arabidopsis* (i.e. *CPKs 1*, *2*, *4*, *5*, *6* and *11*, all of them belonging to cluster I) as sensors and transducers of Ca^2+^ signalling triggered by recognition of pathogen effectors [6,24]. In our study, we found that most of the *CPKs* (such as *MdCPK1b*, *1c*, *4b* and *11*) were differentially expressed in resistant and susceptible cultivars all belong to cluster I, indicating the importance of this cluster in the mechanism of resistance to the *E*. *amylovora* pathogen. Preliminary data has shown down regulation of some *CPK* genes in the flower of susceptible *Malus* after inoculation with *E*. *amylovora*[31].

## Conclusions

Our data can be used to further extend our understanding of the downstream signalling network in fire blight resistant and susceptible apple cultivars by mutant and overexpressing candidate *Malus CPKs* analyses. Since Ca^2+^ and its binding proteins are involved in early recognition of pathogen infection and signal transduction to downstream target molecules [24], it would be interesting to understand downstream target genes and the possible role of phytohormones in regulating pathogen and wound defence mechanisms. We identified a few candidate *CPKs* which are specific to M7 and GD *M*. × *domestica* cultivars. Overexpression or silencing of these *CPKs* might modulate the resistance to *E*. *amylovora* infection. This study provides new tools for clarifying important signalling molecules in regulating the most devastating disease of *Malus* and other Rosaceae host plants.

## Methods

### Plant material and pathogen inoculation

One year old plants of *Malus x domestica* cv Golden Delicious (GD) and own-rooted M.7 rootstock, were grown in the greenhouse at 24°C. *Erwinia amylovora* strain Ea273 was grown overnight at 28°C in Kado medium [59] supplemented with 0.3 g/L MgSO_4_. The inoculum concentration was adjusted to 10^9^ cfu ml^-1^ by dilution with sterile 0.05 M potassium phosphate buffer, pH 6.5. The youngest actively growing leaves of plants were transversally cut using scissors dipped in the bacteria suspension or phosphate buffer as a mechanical damage control [28]. Six plants were inoculated with *Erwinia amylovora* for each time point. Four to six mm wide leaf strips, parallel to the original cut, were collected according to the symptom progression at 0, 2, 6, 12, 24, and 48 hours post inoculation (hpi), frozen in liquid nitrogen and stored at -80°C.

### Database search and identification of *Malus* CPKs

Calcium dependent protein kinase (CPK) genes from *Malus x domestica* were downloaded from the publicly available phytozome (http://www.phytozome.net, http://www.rosaceae.org) database using the hidden Markov model approach as well as the BLASTP protocol [32,33,60]. The BLASTP results are provided in supplementary Additional file 3: Table S1. *CPK* genes from *Arabidopsis thaliana* were used as query sequences to search *Malus CPK* genes. *A*. *thaliana CPK* genes were downloaded from “The *Arabidopsis* Information Resources” (TAIR) (http://www.arabidopsis.org) [44]. All sequences were confirmed by carrying out a BLASTP run against the TAIR database. *Malus x domestica CPKs*, which gave a BLASTP hit with Arabidopsis CPKs, were considered as *Malus CPKs* and the nomenclature was thus carried out accordingly. All the *CPKs* of *M*. *x domestica* were scanned using SCAN PROSITE software to confirm the presence of the EF-hands signature motif and hence *CPK* genes (http://prosite.expasy.org/scanprosite/) [61]. Identified *Malus CPKs* genes were aligned using CLUSTALW software, using BLSOUM62 software with gap open 10, gap extension 0.20, gap distance 5 and clustering neighbour joining [62] to find out the conserved EF-hand domains. Palmytoilation sites of *CPKs* were predicted using CSS palm software [63,64]. The protein sequences were carefully analysed for sequence redundancy followed by removal of alternatively spliced variants. In order to confirm the presence of alternatively spliced gene sequences, the genomic sequence of each candidate gene was also examined. Sequence similarity of *Malus* CPK genes was carried out using online software EMBOSS Needle (http://www.ebi.ac.uk/Tools/psa/emboss_needle/).

### Chromosomal location

The phytozome (http://www.phytozome.net/, http://www.rosaceae.org) database was used for identification of putative *MdCPKs*. Each of the *MdCPKs* was positioned on the *M*. *x domestica* chromosome pseudo molecules using the apple genome browser (http://genomics.research.iasma.it/gb2/gbrowse/apple/).

### Phylogenetic analysis of the *MdCPK* gene family

Multiple sequence alignment analysis carried out using CLUSTALW was used to construct the phylogenetic tree. The *CPKs* of *Oryza sativa*, *A*. *thaliana*, *Populus trichocarpa*, *Zea mays* and *M*. *x domestica* were used to construct the phylogenetic tree with MEGA software, version 5, using the neighbour joining statistical method and Jones-Taylor-Thornton (JTT) model [65].

### RNA isolation and q-PCR

Total RNA from leaves was isolated using the Sigma Spectrum™ plant total RNA kit protocol. Before cDNA synthesis, RNA was treated with RQ1 RNase-free DNase (Promega, Madison, WI, USA) according to the manufacturer’s instructions to ensure no DNA contamination, and first-strand cDNA synthesis was then carried out with approximately 1 μg RNA using an Invitrogen Superscript VILO™ First Strand cDNA Synthesis Kit and oligo-dT primers according to the manufacturer’s procedure. Primers were designed using Primer3 v. 0.4.0 (http://frodo.wi.mit.edu/) with melting temperatures at 58–60°C, primer lengths 20–24 bp and amplicon lengths 250–300 bp. All the primer sequences are listed in Additional file 4: Table S2. q-PCR was conducted on a Biorad iCycler® App 9001 Detection System using SYBR GreenER™ q-PCR supermix (Invitrogen). Reactions were prepared in a total volume of 20 μl containing: 10 μl of 2xSYBR Premix, 2 μl of cDNA template, 0.4 μl of each specific primer to a final concentration of 200 nM. The reactions were performed in the following conditions: initial denaturation step of 95°C for 10 s followed by two-step thermal cycling profile of denaturation at 95°C for 5 s, and combined primer annealing/extension at 60°C for 1 min for 40 cycles. No-template controls were included for each primer pair and each PCR reaction was performed in triplicate on 2 biological replicates. To verify the specificity of the amplicon for each primer pair, melting curve analysis was performed ranging from 60 to 95°C, with temperature increasing steps of 0.06°C/s (five acquisitions per °C) at the end of each run. Baseline and threshold cycles (Ct) were automatically determined using Biorad iCycler® IQ5 Software. Relative expression was calculated as described previously using *EF1* and *UB1* as the reference gene [66,67].

### Determination of intracellular calcium variations using confocal laser scanning microscopy (CLSM) and calcium orange

Calcium orange dye (stock solution in DMSO, Molecular Probes) was diluted in 5 mM MES-Na buffer (pH 6.0) to a final concentration of 5 μM. This solution was applied to intact *M*. *x domestica* leaves as detailed in [68]. Five μM calcium orange solution was applied and after 60 min the leaf was mounted on a Nikon Eclipse C1 spectral CLSM stage, without separating the leaf from the plant, to assess basic fluorescence levels as a control. The microscope operated with a Krypton/Argon laser at 488 nm with a BP of 500–540 nm and a LP of 650 nm. Digital images were analysed using NIH image software as described earlier [53]. After pathogen inoculation (see above) or mechanical damage performed with scissors, leaves were perfused with calcium orange and analysed using CLSM as described above. Controls were represented by application of 5 μM calcium orange solution to intact leaves. At least 5 biological replicates were performed and several images taken for each biological replicate.

### Data and statistical analysis

At least 2 biological replications and 3 technical replication sets were used for the statistical treatment of data. The data are expressed as mean values; error bars indicate the standard error. To evaluate the significance of differences in data, ANOVA followed by Fisher’s PLSD test was performed.

## Abbreviations

CPKs: Calcium-dependent protein kinases; MdCPK: *Malus* x *domestica CPK*; Ca2+: Calcium; [Ca2+]cyt: Cytosolic calcium concentration; Hpi: Hours post inoculation; CaM: Clamodulins; CBL: Calcineurin B-like proteins; MAPK: Mitogen activated protein kinase; MD: mechanical damage.

## Competing interests

The authors declare that there are no competing interests.

## Authors’ contributions

Conception and design of the experiments: CNK, TKM, MM, MEM. Carrying out of experiments: CNK, TKM, AC, AO, FV. Analysis of data: CNK, MEM, MM. Provision of reagents/materials/analysis tools: CNK, MM, MEM. Writing of the paper: CNK, TKM, MEM, MM. All authors read and approved the final manuscript.

## Supplementary Material

Additional file 1: Figure S1 Multiple sequence alignment of *MdCPK* genes. Amino acid sequence alignment of MdCPK genes show presence of kinase domain and four calcium binding EF-hands in regulatory domain. In EF-hands, Ca^2+^ ion are co-ordinated in a pentagonal bipyramidal configuration. Ca^2+^ binding amino acid residue are present at position 1, 3, 5, 7, 9 and 12. The conserved Glu (E) or Asp (D) provides two oxygen for liganding Ca^2+^. Multiple sequence alignment of MdCPK genes were carried out using multalin (http://multalin.toulouse.inra.fr/multalin) software using statistical programme BLOSUM. Red and blue color indicate high and low conserved domains/motifs respectively, whereas black indicate neutral. Click here for file

Additional file 2: Figure S2 Schematic representation of transcript of MdCDPK genes. Box mark represents the exon and line represents the intron of specific CDPK gene. The name to the right of the gene structure indicates the gene name. Click here for file

Additional file 3: Table S1 Q PCR Primer list of all *MdCDPK* genes used in this study. Click here for file

Additional file 4: Table S2 The BLASTP score of MdCPKs found during their identification. The E- value found during BLASTP search show very significant similarity. Click here for file
