# Supplementary figures and images for: Differential expression of CPKs and cytosolic Ca2+ variation in resistant and susceptible apple cultivars (Malus x domestica) in response to the pathogen Erwinia amylovora and mechanical wounding
Source: BMC Genomics. 2013 Nov 5;14:760. doi: 10.1186/1471-2164-14-760 (PMC3840711; doi:10.1186/1471-2164-14-760)

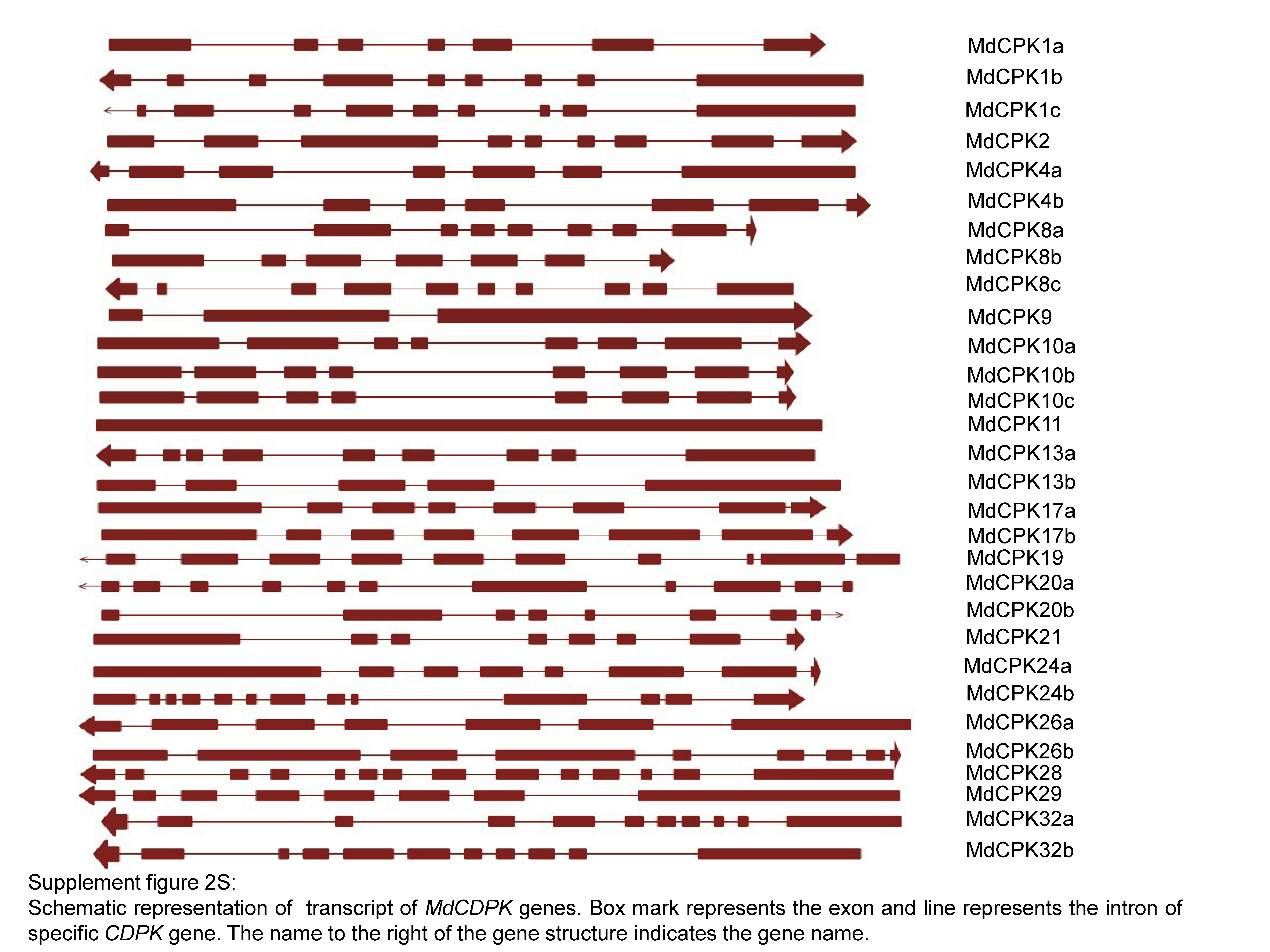

Supplement: Additional file 2: Figure S2 — Schematic representation of transcript of MdCDPK genes. Box mark represents the exon and line represents the intron of specific CDPK gene. The name to the right of the gene structure indicates the gene name. [file 1471-2164-14-760-S2.tiff]
