# Supplementary material for: Differential expression of CPKs and cytosolic Ca2+ variation in resistant and susceptible apple cultivars (Malus x domestica) in response to the pathogen Erwinia amylovora and mechanical wounding
Source: BMC Genomics. 2013 Nov 5;14:760. doi: 10.1186/1471-2164-14-760 (PMC3840711; doi:10.1186/1471-2164-14-760)
Supplement: Additional file 3: Table S1 — Q PCR Primer list of all MdCDPK genes used in this study. [file 1471-2164-14-760-S3.docx]

Name Sequence 5'-3'

MdCPK11-F GACGGACTTCGGTCTCTCTG

MdCPK11-R ATTTTACCGAGCAGGTCACG

MdCPK8a-F TTGAGTGTGGAGTGCTGGAG

MdCPK8a-R GAATTGCTTGAGCCTTGACC

MdCPK13a-F GCAATTCTACGTGGGCTGAT

MdCPK13a-R AATCTGCACCAACCCTCAAG

MdCPK1-F ACCGAGCATTTCCAAGAATG

MdCPK1-R TCGTTTGTAACACCGAACCA

MdCPK26a-F CCATTCACTTGGTGTCATGC

MdCPK26a-R TATCCCTTCAAGACCGCATC

MdCPK26b-F GGAGTTATGCGGATGCATTT

MdCPK26b-R GCAAGCGTCTTGTGAATGAA

MdCPK32a-F ATGATGGACACTGGCAACAA

MdCPK32a-R CTCACCACCACGTCAACATC

MdCPK24a-F CCAGGACAATGCTTGAGGTT

MdCPK24a-R GATAACCCCTGCACTCCAAA

MdCPK1b-F ACCGAGCATTTCCAAGAATG

MdCPK1b-R GCCTGAAGTCCTGCACTAGG

MdCPk17-F GCCACCTCAACTTTTCAAGC

MdCPk17-R CTGATAAGCAACCCGCAAAT

MdCPK1c-F GGACAACGAGAACGTCCATT

MdCPK1c-R GTACGGACTGCCCACAATCT

MdCPK17b-F GACAAGCAGTCCGTCCATTT

MdCPK17b-R GCACTGCCCACAATATCCTT

MdCPK28-F AGGCCACAGACTTTGGTTTG

MdCPK28-R CTTATGCTTGGCCATGGTTT

MdCPK20-F AAAATTGCCATCAGGGTGAG

MdCPK20-R TGCATACAGGTGATCCTCCA

MdCPK32b-F GCTCCTGAGGTGCTTAAACG

MdCPK32b-R TCTTTGCATTTTGCAACCAA

MdCPK24b-F GTTGTTGGATGCTGTGGATG

MdCPK24b-R TCAAAACCTATTCGCCCAAC

MdCPK19-F AATTCGGCGTGACGTATCTC

MdCPK19-R CGAAGAAGTTGCCCTCTCAC

MdCPK29-F GGATGCGAGAAGATGGTGAT

MdCPK29-R CGCTTCCATCAACTGTCTCA

MdCPK8b-F GCGGTTAACAGCTCAGGAAG

MdCPK8b-R CCCAGCTGTTGTATCCCACT

MdCPK9-F GATGCCATATTGGAGGGAGA

MdCPK9-R TTCAGCCCATGGATTTCTTC

MdCPK2-F AGCACCACCAACAATTCACA

MdCPK2-R CAGTCGCTTTCTCCACACAA

MdCPK4-F TTTTGGGCAGGAACTGAATC

MdCPK4-R TTCCGCTATCACACGAAGTG

MdCPK8c-F GAGGGAATTCGGGGTAACAT

MdCPK8c-R ATGACCCCTAGCAACAATGC

MdCPK10-F GCGTTCTGGACTACGGAGAG

MdCPK10-R AGTTCCCGTTTTCATCATCG

MdCPK4b-F ACCTCATGGGCAAGAAATTG

MdCPK4b-R GCGGACGATCCTATCAAAGA

MdCPK9-F TCAACGTTTTCTGGGTCTCC

MdCPK9-R GAGGAGATTTCGGTGCAGAG

MdCPK11-F GTTTTCTACAAGCCCGGTGA

MdCPK11-R GGGCAGTGATTCTCTTCTGC

MdCPK13-F CGATTGATTTCGGCTTGTCT

MdCPK13-R ACTCTTGGCACTCTCCGAAA

MdCPK13b-F CCATTGATTTCGGCTTGTCT

MdCPK13b-R ATCAGCCCACGTAGAATTGC

MdCPK21-F CATCCCAACAACCCCATAAC

MdCPK21-R GCTCACAAGTTTGCGTTTCA

MdCPK20b-F CAAGCGTTGGACTGCAAGTA

MdCPK20b-R CATCCTCATAAGCGCTGACA

Supporting Table S1: Q PCR Primer list of all *MdCDPK* genes used in this study.
