# Supplementary material for: Differential expression of CPKs and cytosolic Ca2+ variation in resistant and susceptible apple cultivars (Malus x domestica) in response to the pathogen Erwinia amylovora and mechanical wounding
Source: BMC Genomics. 2013 Nov 5;14:760. doi: 10.1186/1471-2164-14-760 (PMC3840711; doi:10.1186/1471-2164-14-760)
Supplement: Additional file 4: Table S2 — The BLASTP score of MdCPKs found during their identification. The E- value found during BLASTP search show very significant similarity. [file 1471-2164-14-760-S4.docx]

**Table 2S .** The BLASTP score of MdCPKs found during their identification. The E- value found during BLASTP search show very significant similarity.

| **Sl. No** | **Phytozome Locus ID** | **Gene Name** | **BLASTP Result (Score)** | **E-Value** |
| --- | --- | --- | --- | --- |
| 1 | MDP0000153100 | MdCPK1a | 919.8 | 0 |
| 2 | MDP0000142687 | MdCPK1b | 909.1 | 0 |
| 3 | MDP0000128057 | MdCPK1c | 907.9 | 0 |
| 4 | MDP0000232344 | MdCPK2 | 849.0 | 0 |
| 5 | MDP0000260834 | MdCPK4a | 693.0 | 0 |
| 6 | MDP0000232885 | MdCPK4b | 696.4 | 0 |
| 7 | MDP0000269423 | MdCPK8a | 547.7 | 0 |
| 8 | MDP0000119457 | MdCPK8b | 503.8 | 1.7e-172 |
| 9 | MDP0000260857 | MdCPK8c | 533.9 | 0 |
| 10 | MDP0000169895 | MdCPK9 | 489.6 | 1.4e-166 |
| 11 | MDP0000218522 | MdCPK10a | 547.7 | 0 |
| 12 | MDP0000301254 | MdCPK10b | 556.2 | 0 |
| 13 | MDP0000308706 | MdCPK10c | 556.2 | 0 |
| 14 | MDP0000494270 | MdCPK11 | 679.1 | 0 |
| 15 | MDP0000164868 | MdCPK13a | 551.2 | 0 |
| 16 | MDP0000649496 | MdCPK13b | 298.9 | 1.1e-94 |
| 17 | MDP0000802997 | MdCPK17a | 637.1 | 0 |
| 18 | MDP0000138436 | MdCPK17b | 639.4 | 0 |
| 19 | MDP0000180811 | MdCPK19 | 399.4 | 1.9e-131 |
| 20 | MDP0000318339 | MdCPK20a | 778.9 | 0 |
| 21 | MDP0000513005 | MdCPK20b | 812.0 | 0 |
| 22 | MDP0000232001 | MdCPK21 | 619.8 | 0 |
| 23 | MDP0000262701 | MdCPK24a | 494.6 | 7.3e-168 |
| 24 | MDP0000282003 | MdCPK24b | 485.0 | 7.7e-159 |
| 25 | MDP0000297184 | MdCPK26a | 726.1 | 0 |
| 26 | MDP0000457940 | MdCPK26b | 574.3 | 0 |
| 27 | MDP0000208913 | MdCPK28 | 432.9 | 8.3e-143 |
| 28 | MDP0000142398 | MdCPK29 | 605.1 | 0 |
| 29 | MDP0000649508 | MdCPK32a | 547.4 | 0 |
| 30 | MDP0000179069 | MdCPK32b | 544.3 | 0 |
